# Active trachoma and community use of sanitation, Ethiopia

**DOI:** 10.2471/BLT.16.177758

**Published:** 2017-01-26

**Authors:** William E Oswald, Aisha EP Stewart, Michael R Kramer, Tekola Endeshaw, Mulat Zerihun, Berhanu Melak, Eshetu Sata, Demelash Gessese, Tesfaye Teferi, Zerihun Tadesse, Birhan Guadie, Jonathan D King, Paul M Emerson, Elizabeth K Callahan, Dana Flanders, Christine L Moe, Thomas F Clasen

**Affiliations:** a London School of Hygiene and Tropical Medicine, Keppel Street, London, WC1E 7HT, England.; b The Carter Center, Atlanta, United States of America (USA).; c Rollins School of Public Health, Emory University, Atlanta, USA.; d The Carter Center, Addis Ababa, Ethiopia.; e Amhara Regional Health Bureau, Bahir Dar, Ethiopia.

## Abstract

**Objective:**

To investigate, in Amhara, Ethiopia, the association between prevalence of active trachoma among children aged 1–9 years and community sanitation usage.

**Methods:**

Between 2011 and 2014, prevalence of trachoma and household pit latrine usage were measured in five population-based cross-sectional surveys. Data on observed indicators of latrine use were aggregated into a measure of community sanitation usage calculated as the proportion of households with a latrine in use. All household members were examined for clinical signs, i.e. trachomatous inflammation, follicular and/or intense, indicative of active trachoma. Multilevel logistic regression was used to estimate prevalence odds ratios (OR) and 95% confidence intervals (CI), adjusting for community, household and individual factors, and to evaluate modification by household latrine use and water access.

**Findings:**

In surveyed areas, prevalence of active trachoma among children was estimated to be 29% (95% CI: 28–30) and mean community sanitation usage was 47% (95% CI: 45–48). Despite significant modification (p < 0.0001), no pattern in stratified ORs was detected. Summarizing across strata, community sanitation usage values of 60 to < 80% and ≥ 80% were associated with lower prevalence odds of active trachoma, compared with community sanitation usage of < 20% (OR: 0.76; 95% CI: 0.57–1.03 and OR: 0.67; 95% CI: 0.48–0.95, respectively).

**Conclusion:**

In Amhara, Ethiopia, a negative correlation was observed between community sanitation usage and prevalence of active trachoma among children, highlighting the need for continued efforts to encourage higher levels of sanitation usage and to support sustained use throughout the community, not simply at the household level.

## Résumé

**Objectif:**

Étudier dans la région Amhara, en Éthiopie, l'association entre la prévalence du trachome actif chez les enfants âgés de 1 à 9 ans et l'utilisation d'installations d'assainissement collectif.

**Méthodes:**

Entre 2011 et 2014, la prévalence du trachome et l'utilisation de latrines à fosse domestiques ont été mesurées dans le cadre de cinq enquêtes transversales menées auprès de la population. Les données sur les indicateurs observés concernant l'utilisation de latrines ont été regroupées dans une mesure de l'utilisation des installations d'assainissement collectif exprimée comme la proportion de ménages utilisant des latrines. Tous les membres des ménages ont fait l'objet d'un examen destiné à détecter les signes cliniques, c'est-à-dire une inflammation trachomateuse folliculaire et/ou intense, révélateurs d'un trachome actif. Une régression logistique à plusieurs niveaux a été utilisée pour estimer le rapport de cote (RC) de prévalence ainsi que les intervalles de confiance (IC) à 95%, en tenant compte de facteurs liés à la communauté, aux ménages et aux individus, et pour évaluer les variations induites par l'utilisation de latrines domestiques et un accès à l'eau.

**Résultats:**

Dans les zones étudiées, la prévalence du trachome actif chez les enfants a été estimée à 29% (IC à 95%: 28–30) et l'utilisation moyenne des installations d'assainissement collectif à 47% (IC à 95%: 45–48). Malgré des variations considérables (p < 0,0001), aucune tendance liée aux RC stratifiés ne s'est dégagée. Pour résumer en tenant compte des différentes strates, lorsque les valeurs relatives à l'utilisation des installations d'assainissement collectif étaient comprises entre 60 et 80% ou ≥ 80%, le rapport de cote de prévalence du trachome actif était plus faible que lorsque les valeurs relatives à l'utilisation des installations d'assainissement collectif étaient < 20% (RC: 0,76; IC à 95%: 0,57–1,03 et RC: 0,67; IC à 95%: 0,48–0,95, respectivement).

**Conclusion:**

Dans la région Amhara de l'Éthiopie, une corrélation négative a été observée entre l'utilisation d'installations d'assainissement collectif et la prévalence du trachome actif chez les enfants, soulignant la nécessité de poursuivre les efforts pour encourager l'utilisation d'installations d'assainissement et promouvoir une utilisation durable dans l'ensemble de la population, et non seulement au niveau des ménages.

## Resumen

**Objetivo:**

Investigar, en Amhara, Etiopía, la relación entre la prevalencia del tracoma activo en niños de entre 1 y 9 años y el uso comunitario del saneamiento.

**Métodos:**

Entre 2011 y 2014, se midieron la prevalencia del tracoma y el uso doméstico de las letrinas de pozo en cinco encuestas transversales basadas en la población. Los datos sobre los indicadores observados del uso de letrinas se agregaron en una medida del uso del saneamiento comunitario calculado como el porcentaje de hogares con una letrina en uso. Se examinó a todos los miembros del hogar en busca de síntomas clínicos, es decir, inflamación tracomatosa, folicular y/o intensa, indicio de tracoma activo. Se utilizó una regresión logística de varios niveles para estimar la prevalencia de los cocientes de posibilidades (CP) y los intervalos de confianza (IC) del 95%, ajustándose a factores comunitarios, domésticos e individuales, y para evaluar la modificación por uso de letrinas y acceso al agua en los hogares.

**Resultados:**

En las zonas encuestadas, se estimó que la prevalencia del tracoma activo en niños era del 29% (IC del 95%: 28–30) y que el uso comunitario del saneamiento era del 47% (IC del 95%: 45–48). A pesar de una importante modificación (p < 0,0001), no se detectó ningún patrón en los CP estratificados. Como resumen de los estratos, los valores de uso comunitario del saneamiento del 60 al < 80% y ≥ 80% se relacionaron con una menor prevalencia de tracoma activo, en comparación con un uso comunitario del saneamiento de < 20% (CP: 0,76; IC del 95%: 0,57–1,03 y CP: 0,67; IC del 95%: 0,48-0,95, respectivamente).

**Conclusión:**

En Amhara, Etiopía, se observó una correlación negativa entre el uso comunitario del saneamiento y la prevalencia del tracoma activo en niños, destacando la necesidad de unos esfuerzos continuos por fomentar unos mayores niveles de uso del saneamiento y para respaldar el uso constante en toda la comunidad, no únicamente en los hogares.

## ملخص

**الغرض:**

التحقيق في العلاقة بين انتشار التراخوما النشطة بين الأطفال ممن تتراوح أعمارهم من سنة إلى 9 سنوات واستخدام المرافق الصحية المجتمعية في أمهرة بإثيوبيا.

**الطريقة:**

تم قياس انتشار التراخوما واستخدام المرحاض الحفري المنزلي في خمسة استطلاعات رأي شملت قطاعات متعددة وقائمة على السكان في الفترة ما بين سنة 2011 و2014. وتم تجميع البيانات المتعلقة بالمؤشرات الملحوظة لاستخدام المرحاض للخروج بمقياس لمدى استخدام المرافق الصحية المجتمعية المحتسب كنسبة العوائل التي تستخدم المرحاض. وقد تم فحص جميع أفراد الأسرة بحثًا عن علامات سريرية، أي التهاب تراخومي، أو مسامي، أو حاد، أو مؤشرات تدل على الإصابة بحالة من التراخوما النشطة. وتم استخدام التحوف اللوجيستي متعدد المراحل لتقدير نسب احتمالات الانتشار (OR) ونسب أرجحية تبلغ ‏95‏% (CI)، مع تعديلها حسب العوامل المجتمعية والفردية والأسرية، وتقييم التعديل حسب استخدام العوائل للمراحيض وتوفُّر الماء لديهم.

**النتائج:**

في مناطق الاستطلاع، تم تقدير انتشار التراخوما النشطة بين الأطفال بنسبة ‏29% (95‏% كمقدار لنسبة الأرجحية: 28 – 30) وتقدير متوسط استخدام المرافق الصحية المجتمعية بنسبة 47% (95% كمقدار لنسبة الأرجحية: 45–48). وبالرغم من التعديل الكبير (بمعدل احتمال < 0.0001)، لم يتم الكشف عن نمط معين في نسب احتمالات الانتشار المطبقة. ومن خلال تلخيص البيانات عبر الشرائح المختلفة، فقد تم ربط نسب استخدام المرافق الصحية المجتمعية التي تتراوح من 60 إلى <80‏% و≤ 80‏% باحتمالات منخفضة لانتشار التراخوما، مقارنة باستخدام المرافق الصحية المجتمع بنسبة <20‏% (احتمالات الانتشار: 0.76؛ 95‏% كمقدار لنسبة الأرجحية: 0.57 – 1.03 ونسبة انتشار: 0.67؛ ‏95% كمقدار لنسبة الأرجحية: 0.48 – 0.95، على التوالي).

**الاستنتاج:**

في أمهرة بإثيوبيا، لوحظ وجود ارتباط سلبي بين استخدام المرافق الصحية المجتمعية وانتشار التراخوما النشطة بين الأطفال، مع تسليط الضوء على الحاجة إلى مجهودات مستمرة لتشجيع المستويات الأعلى من استخدام المرافق الصحية، ودعم الاستخدام المستمر عبر المجتمع، وليس على مستوى الأُسرة فحسب. ****

## 摘要

**目的:**

旨在调查埃塞俄比亚阿姆哈拉地区 1 到 9 岁儿童活动性沙眼患病率与社区卫生设施使用情况之间的联系。

**方法:**

2011 年至 2014 年间，五项基于人口的横断面调查对沙眼患病率和家用蹲厕使用情况进行了衡量。 我们汇总了观察到的厕所使用指标数据作为社区卫生设施使用情况的量测指标，按照使用厕所家庭的比例进行计算。 检查了所有家庭成员的临床症状，例如沙眼性炎症、滤泡性和/或严重指示性活动性沙眼。 我们采用多级逻辑回归模型估算了患病率比值比 (OR) 和 95% 置信区间 (CI)，调整了社区、家庭和个人因素，以评估通过家用厕所使用和供水情况进行的整改。

**结果:**

在调查地区，儿童活动性沙眼患病率估计值为 29% (95% CI: 28–30)，社区卫生设施使用率的平均值为 47% (95% CI: 45–48). 除重大调整项目 (p < 0.0001) 以外，未发现分层比值比的模式。 汇总横断面数据得出，与社区卫生设施使用率低于  20% 的情况相比，社区卫生设施使用率在 60% 到 80% 之间和  80% 及以上的情况与较低的活动性沙眼患病率有关联（分别为 OR: 0.76; 95% CI: 0.57–1.03 和 OR: 0.67; 95% CI: 0.48–0.95）。

**结论:**

我们在埃塞俄比亚阿姆哈拉地区观察到，社区卫生设施使用率与儿童活动性沙眼患病率呈负相关，突出了需要继续努力促进更高水平的卫生设施使用率并支持在整个社会的持续使用，而不仅限于家庭层面上。

## Резюме

**Цель:**

Изучить в регионе Амхара, Эфиопия, связь между распространенностью активной трахомы среди детей в возрасте 1–9 лет и общинным использованием санитарно-гигиенических удобств.

**Методы:**

В период с 2011 по 2014 год в ходе пяти поперечно-секционных обследований популяционного масштаба была изучена распространенность трахомы и использования ямного туалета домашними хозяйствами. Данные по наблюдаемым показателям использования ямных туалетов были объединены, чтобы получить масштаб общинного использования санитарно-гигиенических удобств, выраженный долей домашних хозяйств, в которых используются ямные туалеты. Все члены семей были обследованы на предмет клинических признаков, т. е. трахоматозного воспаления, фолликулярного и (или) интенсивного, указывающего на активную трахому. Многоуровневая логистическая регрессия была использована для оценки отношений шансов (ОШ) и 95%-х доверительных интервалов (ДИ) распространенности с учетом общинных, бытовых и индивидуальных факторов, а также для оценки влияния использования ямного туалета и доступа к воде в домашнем хозяйстве.

**Результаты:**

В обследованных районах распространенность активной трахомы среди детей оценивалась как 29% (95%-й ДИ: 28–30) и среднее значение использования санитарии общиной составляло 47% (95%-й ДИ: 45–48). Несмотря на значительное влияние (р < 0,0001), не было обнаружено никакой закономерности в стратифицированных ОШ. Подводя итог по стратам, можно отметить, что там, где показатели использования санитарно-гигиенических удобств общиной составляли от 60 до <80% и ≥ 80%, наблюдалась более низкая вероятность распространенности активной трахомы по сравнению со стратой, где показатель использования санитарно-гигиенических удобств общиной составил менее 20% (ОШ: 0,76; 95%-й ДИ: 0,57–1,03 и ОШ: 0,67; 95%-й ДИ: 0,48–0,95 соответственно).

**Вывод:**

В регионе Амхара, Эфиопия, наблюдалась отрицательная корреляция между общинным использованием санитарно-гигиенических удобств и распространенностью активной трахомы среди детей, что указывает на необходимость в непрекращающихся мерах по стимулированию более высокого уровня использования санитарно-гигиенических удобств и поддержке постоянного использования всей общиной, а не только на уровне домашнего хозяйства.

## Introduction

It has been estimated that, as a result of trachoma, approximately 1.2 million people are blind and a further 1.7 million have low vision.^1^ Globally, trachoma remains the leading infectious cause of blindness. In 2009, an estimated 40.6 million people had active trachoma and 8.2 million had trichiasis – i.e. the blinding stage of the disease.^2^ About 77% of those living in trachoma-endemic areas of the world are to be found in 29 of the countries in the World Health Organization’s (WHO’s) African Region, and Ethiopia is the country most affected by trachoma worldwide.^3^ Trachoma is caused by ocular infection with a bacterium: *Chlamydia trachomatis*. Inflammation attributable to repeat infections during childhood constitutes the disease’s active stage. This inflammation may then lead to scarring of the conjunctiva and trichiasis. In trichiasis, the eyelashes rub and damage the cornea, causing pain and, eventually, blindness. Trachoma is predominantly found in resource-poor, rural communities in low-income countries.^4^^,^^5^ By afflicting some of the most deprived people in the world, it leads to disability, dependency and further poverty.^6^

A pilot programme for trachoma control, begun in four districts of the Amhara region in north-west Ethiopia, was scaled-up so that the programme covered the whole of Amhara by 2007.^7^^,^^8^ This ongoing programme, based on WHO’s SAFE strategy, was set in Amhara because this region has the highest burden of active trachoma within Ethiopia.^9^ The SAFE strategy is a comprehensive WHO strategy – based on the available relevant biological and epidemiological evidence – to treat and prevent trachoma. It combines four measures: (i) surgery for the correction of trichiasis; (ii) antibiotics, given in mass drug administrations, to reduce the infection reservoir in the community; (iii) facial cleanliness, to reduce transmission; and (iv) environmental improvements – e.g. control of flies through sanitation and improved access to water for hygiene – for further reductions in the potential for transmission.^10^^,^^11^

The presence of latrines or other facilities for the disposal of human faeces is understood to have an indirect beneficial effect on the risk of trachoma because it reduces the access of *Musca sorbens* flies – a probable vector of *C. trachomatis* – to potential breeding sites.^12^^–^^14^ As flies can easily move throughout an area, however, a few scattered latrines may have little impact on trachoma in that community – or even in households with latrines.^6^ Effective fly control through sanitation requires not only high levels of latrine access but also consistent latrine use throughout the community.^15^ Programmes to improve sanitation and/or control trachoma typically measure changes in sanitation at regional or national level. If they do investigate latrine coverage at community level at all, they tend to record latrine access rather than use. The role of latrine use – at community level – in trachoma control requires elucidation.^16^

We believed that, rather than latrine access at household level, the proportion of households in a community with latrines in use would be a stronger indicator of the effectiveness of fly control and levels of exposure to *C. trachomatis*.^15^ We hypothesized that higher community sanitation usage would be associated with a lower prevalence of active trachoma.

## Methods

### Study overview and subjects

Between 2011 and 2014, the Amhara Regional Health Bureau conducted trachoma-impact surveys in various areas of Amhara. These surveys were designed to provide population-based estimates of trachoma prevalence, quantify uptake of trachoma control efforts and estimate the proportions of households with water and sanitation access.^17^ A district – known locally as a *woreda –* only became eligible for surveying when at least five annual rounds of mass administrations of azithromycin had occurred. Each survey was conducted at least six months after the last such antibiotic administration in the target district.

For the present study, we combined data collected in trachoma-impact surveys that were conducted in the South Gondar zone in June–August 2011,^18^^,^^19^ in the North Gondar and West Gojjam zones in May–June 2012, in eastern Amhara between December 2012 and January 2013, in western Amhara in June–July 2013, and in eastern Amhara in January–February 2014.

All five surveys used multistage cluster random sampling to estimate the district-level prevalence of trachomatous inflammation–follicular. Villages – known locally as *gott* – represented the smallest administrative unit with available population data and were the primary sampling units. Within each target district, villages were selected, from a geographically-ordered listing, using probability-proportional-to-size sampling. Within each selected village, smaller administrative units of approximately 40 households – i.e. household clusters that were locally called development teams – were used as segments for a modified segment design.^20^^,^^21^ Such clusters were listed and given identification numbers upon arrival in a study village with the assistance of a designated village representative, who then drew a number from a hat to select the cluster to be surveyed. The entire village was surveyed if it consisted of 40 or fewer households.

Community information was collected in interviews with village leaders. In each selected cluster, all residents who gave verbal consent were examined for clinical signs of trachoma, according to WHO guidelines.^17^ Each eye was examined separately, by a trained trachoma grader using a 2.5x  binocular loupe, for the presence or absence of all five clinical signs of the simplified trachoma grading system.^22^ Heads of household were interviewed for demographic and socioeconomic information as well as knowledge and practices regarding trachoma, hygiene, sanitation and water. Visual inspections were made of household latrines and hand-washing stations. Responses were recorded electronically using tablet computers with Swift Insights software (The Carter Center, Atlanta, USA). Questionnaires at community, household and individual levels were linked.^23^

### Measures

The exposure variable – community sanitation usage – was calculated as the proportion of households within the cluster with a latrine with evidence of use. A latrine was considered to be in use if there was a defined path to it and faeces were observed in the pit.^24^

For trachoma, the outcome variable was a dichotomous measure, based on WHO’s simplified grading scale, for the absence/presence of active trachoma – i.e. absence/presence of trachomatous inflammation – follicular and/or intense.^17^

### Analyses

We used multilevel logistic regression to estimate the association between the proportion of households in each cluster with a latrine in use and active trachoma among children aged 1–9 years – accounting for dependence of observations nested within households and clusters. Multilevel analysis can assess the influence of area-level effects – e.g. community sanitation usage – on an individual outcome in addition to between-group and within-group variability.^25^ These variance-based measures provide a useful complement to standard measures of association for the analysis of contextual effects.^26^

Accounting for study design and unequal selection probabilities, means and proportions were estimated, with 95% confidence intervals (CI), across categories of community sanitation usage. Generalized linear mixed models were fitted, specifying random intercepts for cluster and households nested within clusters. Models were estimated using adaptive quadrature with eight integration points, and robust standard errors (SE) were requested to account for clustering within districts. Sampling weights, based on the inverse selection probability for cluster, household and individual, were incorporated. Individual and household weights were scaled to sum to the household and cluster sample size, respectively.^27^ After scaling weights, to restrict analysis to the subpopulation of interest, participants who were not aged 1–9 years were assigned an individual weight of 0.0001.^28^ Our treatment of the exposure variable as a categorical measure – rather than a linear or quadratic measure – was based on the results of a preliminary assessment that considered both fit and interpretability. An empty model was fitted to measure between-cluster and between-household variance.^29^ Potential confounders were identified, from the community-level, household and individual covariates recorded in the surveys (Table 1), based on the results of a literature review, an evaluation of directed acyclic graphs,^31^^,^^32^ univariable analyses and initial unweighted modelling. We used a sequential modelling approach to explore confounding –indicated by change in exposure estimates – and changes in residual variance. All models controlled for survey round, to account for year and possible between-survey variations in the method. Most surveys were conducted during the rainy season, so an assessment of seasonality was not possible. We calculated intraclass correlation coefficients by converting individual-level and area-level components of variance to the same scale – using the latent variable method – and median odds ratios (ORs) were calculated as measures of residual variance on the OR scale.^29^^,^^33^ To evaluate multiplicative modification of the effect of community sanitation usage on active trachoma, by household latrine use and water access, we used likelihood ratio tests, after controlling for covariates included in the fully-adjusted model. We calculated summary measures of association with CI from the weighted means of stratum-specific log ORs.^34^^,^^35^ Models included all observations with information available for included covariates. Initial unweighted modelling was conducted in SAS 9.4 (SAS Institute, Cary, United States of America). All described analyses were conducted using Stata version 13.1 (StataCorp. LP, College Station, USA).

**Table 1:** Active trachoma prevalence and community, household and individual characteristics of children aged 1–9 years with eye examination results, categorized according to community sanitation usage, Amhara, Ethiopia, 2011–2014

Characteristics	*n*	Value (95% CI) for clusters where percentage of households with latrines with evidence of use was:^a^					
		0 to < 20	20 to < 40	40 to < 60	60 to < 80	≥ 80	All
**% of children**							
With active trachoma	62 869	0.31(0.29–0.33)	0.33 (0.30–0.36)	0.30 (0.28–0.33)	0.26 (0.23–0.28)	0.23 (0.21–0.25)	0.29 (0.28–0.30)
With trachomatous inflammation							
Follicular	62 869	0.28 (0.26–0.31)	0.30(0.27–0.34)	0.28 (0.25–0.31)	0.23 (0.21–0.25)	0.21 (0.19–0.23)	0.26 (0.25–0.27)
Intense	62 869	0.06 (0.05–0.07)	0.06 (0.05–0.07)	0.07(0.05–0.08)	0.05 (0.04–0.06)	0.04 (0.04–0.05)	0.06 (0.05–0.06)
**Community**							
No. of children’s communities	1 510	412	279	260	302	257	1 510
Median no. of MDA received^b^	1 510	2.37 (2.28–2.47)	2.62 (2.53–2.71)	2.72 (2.62–2.83)	2.72 (2.63–2.81)	2.91 (2.80–3.02)	2.65 (2.61–2.69)
People per square kilometer^c^	1 508	303 (206–399)	391 (280–501)	665 (348–982)	1 022 (701–1 343)	1 696 (1 244–2 149)	786 (670–901)
Proportion of communities with health facility^d^	1 414	0.15 (0.11–0.20)	0.28 (0.22–0.35)	0.28 (0.22–0.35)	0.26 (0.21–0.32)	0.33 (0.27–0.39)	0.25 (0.23–0.28)
No. of wealth indicators per household^e^	1 510	0.74 (0.68–0.79)	0.95 (0.87–1.03)	1.19 (1.09–1.28)	1.38 (1.27–1.49)	1.72 (1.58–1.87)	1.17 (1.13–1.21)
**Household**							
No. of children’s households	35 977	10 279	6 764	6 070	7 043	5 821	35 977
% of households In which highest education of any adult was:^f^	35 595	**–**	**–**	**–**	**–**	**–**	**–**
None	**–**	0.56 (0.54–0.59)	0.53 (0.49–0.56)	0.49 (0.45–0.52)	0.43 (0.40–0.46)	0.43 (0.39–0.47)	0.49 (0.48–0.51)
Religious	**–**	0.02 (0.02–0.03)	0.03 (0.02–0.04)	0.03 (0.02–0.03)	0.03 (0.02–0.04)	0.02 (0.02–0.03)	0.03 (0.02–0.03)
Primary school	**–**	0.21 (0.19–0.23)	0.16 (0.14–0.18)	0.19 (0.16–0.21)	0.18(0.16–0.20)	0.18 (0.16–0.21)	0.18 (0.18–0.19)
Junior secondary	**–**	0.13 (0.11–0.14)	0.17 (0.15–0.19)	0.17 (0.15–0.19)	0.19 (0.17–0.20)	0.17 (0.15–0.19)	0.16 (0.16–0.17)
Senior secondary	**–**	0.04 (0.03–0.04)	0.06 (0.05–0.08)	0.08 (0.07–0.09)	0.11 (0.10–0.12)	0.12 (0.10–0.14)	0.08 (0.07–0.08)
College or university	**–**	0.00 (0.00–0.01)	0.01 (0.01–0.01)	0.01 (0.01–0.02)	0.03 (0.02–0.04)	0.04 (0.03–0.05)	0.02 (0.02–0.02)
Non-formal	**–**	0.04(0.03–0.05)	0.04 (0.03–0.05)	0.04 (0.03–0.05)	0.04 (0.03–0.05)	0.03 (0.02–0.04)	0.04 (0.03–0.04)
% of households owning mobile phone	35 584	0.11 (0.09–0.13)	0.18 (0.15–0.21)	0.23 (0.20–0.26)	0.25 (0.21–0.28)	0.32 (0.29–0.36)	0.21 (0.19–0.22)
% of households owning radio	35 599	0.11 (0.10–0.13)	0.13 (0.11–0.15)	0.18 (0.16–0.20)	0.22 (0.19–0.24)	0.24 (0.21–0.27)	0.17 (0.16–0.18)
% of households owning television	35 589	0.00 (0.00–0.01)	0.01 (0.01–0.02)	0.02 (0.01–0.03)	0.05 (0.03–0.08)	0.12 (0.09–0.14)	0.04 (0.03–0.04)
% of households with bathing water within a journey of < 30 minutes	37 502	0.61 (0.57–0.66)	0.69 (0.64–0.73)	0.68 (0.62–0.73)	0.70 (0.65–0.74)	0.74 (0.69–0.78)	0.68 (0.66–0.70)
% of households with a latrine	35 863	0.12 (0.11–0.14)	0.38 (0.36–0.39)	0.58 (0.56–0.59)	0.77 (0.76–0.78)	0.93 (0.92–0.94)	0.52 (0.50–0.53)
% of households with a latrine with evidence of use	35 762	0.08 (0.07–0.09)	0.32(0.31–0.33)	0.53 (0.52–0.54)	0.74 (0.73–0.75)	0.92 (0.91–0.93)	0.48 (0.47–0.50)
% of households with any trachoma prevention knowledge^g^	35 565	0.52 (0.48–0.55)	0.58 (0.53–0.62)	0.60 (0.55–0.64)	0.70 (0.66–0.72)	0.70 (0.66–0.73)	0.61 (0.60–0.62)
% of households with electricity	35 584	0.02 (0.01–0.04)	0.06 (0.03–0.09)	0.08 (0.05–0.12)	0.18 (0.14–0.23)	0.26 (0.21–0.31)	0.11 (0.10–0.13)
% of households with iron roof	35 782	0.51 (0.48–0.55)	0.58 (0.53–0.62)	0.67 (0.62–0.71)	0.73 (0.70–0.77)	0.75 (0.71–0.79)	0.64 (0.62–0.65)
**Individual**							
No. of children	62 869	19 484	11 755	10 162	11 832	9 636	62 869
Age in years	62 869	4.99 (4.94–5.04)	5.10 (5.04–5.17)	5.15 (5.09–5.22)	5.18 (5.12–5.25)	5.23 (5.15–5.30)	5.12 (5.09–5.14)
% being boys	62 806	0.48 (0.47–0.49)	0.48 (0.47–0.49)	0.49 (0.47–0.50)	0.49 (0.48–0.50)	0.48 (0.47–0.49)	0.48 (0.48–0.49)
Reporting receipt of:	62 158						
0 MDA		0.26 (0.23–0.29)	0.22 (0.19–0.24)	0.19 (0.16–0.21)	0.18 (0.15–0.21)	0.15 (0.13–0.17)	0.21 (0.19–0.22)
1 MDA		0.15 (0.14–0.16)	0.15 (0.14–0.17)	0.15 (0.14–0.16)	0.16 (0.14–0.17)	0.14 (0.13–0.15)	0.15 (0.14–0.16)
2 MDA		0.32 (0.30–0.34)	0.33 (0.31–0.35)	0.31 (0.29–0.34)	0.32 (0.30–0.34)	0.29 (0.27–0.32)	0.32 (0.31–0.33)
3 MDA		0.22 (0.20–0.24)	0.24 (0.22–0.27)	0.26 (0.24–0.29)	0.26 (0.24–0.29)	0.30 (0.27–0.32)	0.25 (0.24–0.26)
4 MDA		0.04 (0.03–0.05)	0.05 (0.04–0.07)	0.07 (0.06–0.09)	0.07 (0.05–0.08)	0.10 (0.08–0.12)	0.06 (0.06–0.07)
5 MDA		0.01 (0.01–0.02)	0.01 (0.00–0.01)	0.01 (0.01–0.02)	0.02 (0.01–0.03)	0.03 (0.02–0.04)	0.01 (0.01–0.02)
Reporting receipt of at least one MDA	62 767	0.74 (0.72–0.77)	0.79 (0.76–0.81)	0.82 (0.79–0.84)	0.82 (0.79–0.85)	0.85 (0.83–0.88)	0.80 (0.78–0.81)
Reporting school attendance	61 185	0.17 (0.16–0.19)	0.21 (0.20–0.23)	0.24 (0.22–0.26)	0.25 (0.24–0.27)	0.29 (0.28–0.31)	0.23 (0.22–0.23)
Without ocular or nasal discharge	58 450	0.72 (0.69–0.75)	0.75 (0.72–0.78)	0.79 (0.76–0.82)	0.77 (0.74–0.80)	0.82 (0.80–0.85)	0.77 (0.75–0.78)

CI: confidence interval; MDA: mass drug administration.

^a^ All values followed by confidence intervals are means.

^b^ Reportedly, by all residents.

^c^ Values extracted, using geographical coordinates in ArcMap 10.1 (ESRI, Redlands, USA), from raster surface with 2011 population density, generated using the Oak Ridge National Laboratory’s LandScan.^30^

^d^ A health centre, health post or hospital.

^e^ The household wealth indicators were ownership of mobile phone, ownership of radio, ownership of television, an iron roof and access to electricity.

^f^ The highest level of education reported to have been completed either by respondents in the 2011 survey or by any household member in the subsequent surveys.

^g^ That is, the unprompted reporting of one or more forms of trachoma prevention.

### Ethical approval

The protocols for the surveys that were our data sources were approved by Emory University’s Institutional Review Board and the Amhara Regional Health Bureau. Our secondary analysis was exempt from additional review.

## Results

Of 56 425 households surveyed in 1510 clusters throughout Amhara region, 56 169 (> 99%) were linked to eye examination and census information, for 233 363 individuals. Of 68 961 children aged 1–9 years in the linked data set, 62 869 (91%), in 35 977 households, had eye examination results. Of 6092 children aged 1–9 years in the linked data set that lacked eye examination results, 4864 (80%) were reported to be out, travelling or at school during the survey, 734 (12%) refused the examination and 494 (8%) did not have a reason provided. Community sanitation usage was calculated using data on 56 050 (> 99%) of the surveyed households.

Table 1 summarizes overall, and by category of community sanitation usage, the community, household and individual characteristics of children aged 1–9 years with eye examination results. In general, compared with other children, those in communities with relatively low sanitation usage had indicators of poorer hygiene, more impoverished and rural living conditions and less education and health care. In terms of mean household counts of wealth indicators, communities with the lowest category of sanitation usage appeared much poorer than communities with the highest category of such usage (Table 1).

Levels of exposure to mass administrations of antibiotic for trachoma control and levels of prevention knowledge mirrored patterns of community sanitation usage. For example, children in communities with the lowest category of sanitation usage were less likely to have ever received antibiotics during mass administrations than children in communities with the highest category of such usage (Table 1). The median number of times that all community residents had reportedly received such antibiotics was lower in communities with the lowest category of sanitation usage than in communities with the highest category of usage. Some trachoma prevention knowledge was reported by more than half of households (Table 1) but such knowledge was less frequently reported in communities with relatively low sanitation usages. Where latrines were present, latrine usage was high. Among households, an estimated 52% (95% CI: 50–53) owned a pit latrine, the primary form of sanitation recorded. Of these pit latrines, 93% (95% CI: 92–93) were classified as in use based on observation. The mean reported age of latrines was 2.59 years (95% CI: 2.49–2.70), based on data from three of the surveys that recorded such data.

Mean community sanitation usage was 47% (95% CI: 45–48) over all 1510 clusters but ranged from 0% in 106 clusters to 100% in 25 clusters. The overall prevalence of active trachoma in children aged 1–9 years was estimated to be 29% (95% CI: 28–30) (Table 1).

We fitted several models, described as models 1–6, to examine the association between community sanitation usage and active trachoma – sequentially controlling for selected community, household and individual factors and adjusting for survey (Table 2). In terms of the available covariates, populations for all six models were similar. In model 1, which only adjusted for survey round, community sanitation usages of 60 to < 80% and ≥ 80% were associated with lower prevalence odds of active trachoma compared with usage of < 20%. Adding adjustments for child’s age and sex (model 2) and then household water access and latrine use (model 3) did not meaningfully change the estimated ORs. After inclusion of household wealth indicators and education (model 4), an aggregated community wealth measure (model 5) and a community measure for the median number of times that a mass antibiotic administration had been received (model 6), the pattern remained the same but was attenuated towards null (Table 2).

**Table 2:** Results from six models of the association between active trachoma in children aged 1–9 years and community sanitation usage, Amhara, Ethiopia, 2011–2014

Characteristics	Model^a^					
	1	2	3	4	5	6
**No. of individuals**	62 869	62 806	62 037	61 351	61 351	61 351
**No. of households**	35 977	35 963	35 477	35 061	35 061	35 061
**No. of clusters of households**	1 510	1 510	1 510	1 510	1 510	1 510
**aOR (95% CI) with community sanitation usage of:^b^**						
≥ 80%	0.51 (0.35–0.73)	0.50 (0.33–0.75)	0.54 (0.36–0.81)	0.62 (0.42–0.92)	0.74 (0.49–1.11)	0.68 (0.45–1.04)
60 to < 80%	0.67 (0.47–0.97)	0.68 (0.45–1.03)	0.73 (0.49–1.10)	0.78 (0.52–1.17)	0.88 (0.58–1.32)	0.84 (0.55–1.26)
40 to < 60%	0.91 (0.66–1.26)	0.95 (0.66–1.35)	1.00 (0.70–1.42)	1.02 (0.72–1.46)	1.11 (0.78–1.58)	1.06 (0.74–1.52)
20 to < 40%	1.03 (0.76–1.39)	1.07 (0.77–1.49)	1.10 (0.79–1.53)	1.11 (0.80–1.54)	1.16 (0.83–1.60)	1.12 (0.81–1.55)
< 20%	Ref.	Ref.	Ref.	Ref.	Ref.	Ref.
**Random effects**						
Intraclass correlation coefficient						
Same cluster	0.34	0.37	0.37	0.36	0.36	0.35
Same household	0.45	0.52	0.52	0.51	0.51	0.51
Median OR						
Cluster	4.80	6.07	6.02	5.86	5.83	5.82
Households within cluster	2.19	2.66	2.65	2.63	2.63	2.63
Variance between clusters (SE)	2.03 (0.18)	2.52 (0.23)	2.50 (0.22)	2.41 (0.22)	2.39 (0.22)	2.38 (0.22)
Variance between households (SE)	0.68 (0.08)	1.05 (0.10)	1.04 (0.10)	1.03 (0.10)	1.03 (0.10)	1.03 (0.10)

aOR: adjusted odds ratio; CI: confidence interval; OR: odds ratio; Ref.: reference group; SE: standard error.

^a^ Results were weighted to account for the unequal probabilities of selection and down-weight survey participants excluded from the assessment of trachoma prevalence because they were aged < 1 year or > 9 years. Model 1 was adjusted for survey round. Model 2 was like model 1 but also adjusted for sex and age of the child– with age centred at 5 years. Model 3 was like model 2 but also adjusted for household access to bathing water within a journey of < 30 minutes and household latrine with evidence of use. Model 4 was like model 3 but also adjusted for household access to electricity, household education, household ownership of mobile phone, radio and/or television and iron roof. Model 5 was like model 4 but also adjusted for community mean household wealth. Model 6 was like model 5 but also adjusted for the median number of times surveyed residents in a community had reportedly received a mass drug administration against trachoma.

^b^ Usage was measured as the proportion of households with a latrine with evidence of use.

In the empty model, which excluded any covariates, residual variances between households and between clusters were estimated to be 0.68 (SE: 0.08) and 2.22 (SE: 0.20), respectively. Intraclass correlation coefficients for the same household and for different households in the same cluster were calculated as 0.47 and 0.36, respectively. In this study, median ORs indicate the extent, always greater than or equal to 1, to which the individual probability of active trachoma was determined by cluster and household levels. In a comparison of children of different households from different communities, the median OR was calculated to be 5.07. In a comparison of children of different households from the same community, the corresponding ratio was 2.19. Variance did not meaningfully change across additional models (Table 2). Residual heterogeneity between clusters was of greater relevance than community sanitation usage. In model 6, median ORs indicated that residual heterogeneity between children of different communities reflected, on average, a 5.82-fold increase in the individual odds of active trachoma.

The magnitude of association between community sanitation usage and active trachoma was found to vary significantly by household latrine use and water access (*P* < 0.0001). As no clear pattern in stratified OR estimates and CIs was discerned, summary estimates, weighted by population in the strata of household latrine use and water access, are reported by category of community sanitation usage in Table 3.

**Table 3:** Modification by household latrine use and water access of the association between active trachoma in children aged 1–9 years and community sanitation usage, Amhara, Ethiopia, 2011–2014

Community sanitation usage (% of households with latrines in apparent use)	Household owns latrine with evidence of use						Household does not own latrine with evidence of use^a^					*n*	sOR (95% CI)^b^
	Water access < 30 minutes			Water access ≥ 30 minutes			Water access < 30 minutes			Water access ≥ 30 minutes			
	*n*	OR (95% CI)		*n*	OR (95% CI)		*n*	OR (95% CI)		*n*	OR (95% CI)		
≥ 80	6 383	0.62 (0.39–0.99)		2 332	0.51 (0.32–0.83)		518	0.68 (0.41–1.13)		182	0.93 (0.52–1.67)	9 415	0.67 (0.48–0.95)
60 to < 80	5 870	0.83 (0.54–1.29)		2 690	0.54 (0.32–0.90)		2 006	0.87 (0.55–1.37)		982	0.68 (0.42–1.09)	11 548	0.76 (0.57–1.03)
40 to < 60	3 636	0.87 (0.56–1.36)		1 741	0.76 (0.46–1.24)		3 094	1.10 (0.75–1.62)		1 471	1.35 (0.92–1.96)	9 942	1.01 (0.74–1.37)
20 to < 40	2 587	1.14 (0.75–1.72)		1 237	0.72 (0.46–1.13)		5 282	1.07 (0.74–1.56)		2 392	1.22 (0.87–1.69)	11 498	1.06 (0.78–1.44)
< 20	1 090	Ref.		492	Ref.		10 679	Ref.		6 687	Ref.	18 948	Ref.

CI: confidence interval; OR: odds ratio; Ref.: reference group; sOR: summary odds ratio.

^a^ Because the household has no latrine or has a latrine considered to be unused.

^b^ Summary OR weighted by total population within strata of household water and sanitation access, adjusted for age, sex, household education, household wealth items, community mean sum of household wealth indicators, median number of times surveyed residents in a community had reportedly received a mass drug administration against trachoma and survey round.

## Discussion

Our study shows that increasing the proportion of households in a community with latrines in use may be protective against active trachoma among children aged 1–9 years, independent of whether a child’s household had a latrine in use or better access to water and controlling for potential confounders. There was no clear evidence of multiplicative modification of the effect of community sanitation usage on active trachoma by household latrine use and water access. Multilevel analysis, which allowed estimation of residual variation between communities and households, indicated the importance of additional contextual factors – beyond community sanitation usage and other measures that we included in our models – that may have more influence on an individual’s propensity for active trachoma.

Studies in the Gambia identified the fly, *M. sorbens* – that breeds in openly-deposited faeces of humans and other mammals but not in pit latrines – as an insect vector of trachoma, clarifying the relationship between faeces in the environment and the disease.^12^^–^^14^ Subsequently, in a randomized controlled trial, fly catches from children’s eyes and mean active trachoma prevalence were reduced through latrine provision – but not by a statistically significant amount.^36^ In another randomized controlled study – designed to measure the effect of latrine promotion on re-emergence of trachoma after a mass administration of antibiotic – there was no evidence of a significant relationship between increased latrine provision and prevalence of active trachoma or *C. trachomatis* infection in children because there was no rapid re-emergence of infection in either study arm.^37^ In a later cohort analysis of the communities that had received a single mass azithromycin distribution and promotion of latrine usage, it was found that, for each 10% increase in the proportion of household latrines with evidence of use 12 months after baseline, there was a 2.0% decrease (95% CI: 0.2–3.9) in the community prevalence of ocular *C. trachomatis* infection over the subsequent year.^38^ However, no corresponding decrease in the prevalence of active trachoma was observed,^38^ perhaps because the follow-up period was too short to allow the beneficial impact of a cleaner living environment on the occurrence of active trachoma to become apparent.^39^ Based on available data, most latrines observed in the surveys we used had been in place for more than 12 months. 

In the control of neglected tropical diseases, the relative importance of hygiene, sanitation and water components, and of household sanitation compared with community sanitation, remains to be established.^16^^,^^40^ Improvements in access to water supplies could lead to increased facial cleansing, one of the four components of the SAFE strategy, by increasing the quantity of water available at household level. However, we did not identify any pattern of difference in the association between community sanitation usage and active trachoma by household water access and latrine usage, despite statistically significant interaction.

Few studies have examined the relationship between community sanitation usage and health outcomes.^16^^,^^41^^–^^44^ The conclusions from this study are strengthened by its size, population-based estimates and consideration of latrine use rather than latrine ownership. Our study had limitations. The cross-sectional surveys prevent causal conclusions, and residual confounding remains possible. We could not control for hygiene practices because few relevant measures were collected in the surveys. Also, our indicator of latrine use did not measure usage by all household members or the disposal of children’s faeces. Therefore, we had to assume that the proportion of households with a latrine with evidence of use reflected the actual proportion of the community population that consistently deposited their faeces in a latrine.^24^

Although we observed variation in the prevalence of active trachoma at district-level, our estimate of the overall prevalence of active trachoma among children aged 1–9 years in Amhara, i.e. 29%, indicates the need for continued local implementation of the SAFE strategy. At the time of our study, despite improvements over recent years across Ethiopia,^45^ household latrine usage in Amhara remained below 50%. Trachoma control efforts should continue to emphasize environmental improvements. The association of community sanitation usage with trachoma highlights the need for interventions – particularly ones targeting the environmental component of the SAFE strategy – to create communities free from open defecation.^46^ By modelling the association between community sanitation usage and *C. trachomatis* infection, it might be possible to clarify the role of sanitation in preventing transmission of the causative agent of trachoma. Future research should focus on both increasing the adoption of latrines – to reach protective levels of community sanitation usage – and improving latrine construction and maintenance – to ensure that any usage improvements are sustained.
